# Improvements of the Hermite-Hadamard inequality for the simplex

**DOI:** 10.1186/s13660-016-1273-z

**Published:** 2017-01-03

**Authors:** Zlatko Pavić

**Affiliations:** Department of Mathematics, Mechanical Engineering Faculty in Slavonski Brod, University of Osijek, Slavonski Brod, 35000 Croatia

**Keywords:** 26B25, 52A40, convex combination, simplex, the Hermite-Hadamard inequality

## Abstract

In this study, the simplex whose vertices are barycenters of the given simplex facets plays an essential role. The article provides an extension of the Hermite-Hadamard inequality from the simplex barycenter to any point of the inscribed simplex except its vertices. A two-sided refinement of the generalized inequality is obtained in completion of this work.

## Introduction

A concise approach to the concept of affinity and convexity is as follows. Let $\mathbb{X}$ be a linear space over the field $\mathbb{R}$. Let $P_{1},\ldots,P_{m}\in\mathbb{X}$ be points, and let $\lambda_{1},\ldots ,\lambda_{m}\in\mathbb{R}$ be coefficients. A linear combination

1 $$ \sum_{j=1}^{m} \lambda_{j}P_{j} $$

is affine if $\sum_{j=1}^{m}\lambda_{j}=1$. An affine combination is convex if all coefficients $\lambda_{j}$ are nonnegative.

Let $\mathcal{S}\subseteq\mathbb{X}$ be a set. The set containing all affine combinations of points of $\mathcal{S}$ is called the affine hull of the set $\mathcal{S}$, and it is denoted with $\operatorname{aff}\mathcal{S}$. A set $\mathcal{S}$ is affine if $\mathcal{S}=\operatorname{aff}\mathcal{S}$. Using the adjective convex instead of affine, and the prefix conv instead of aff, we obtain the characterization of the convex set.

A convex function $f:\operatorname{conv}\mathcal{S}\to\mathbb{R}$ satisfies the Jensen inequality

2 $$ f \Biggl(\sum_{j=1}^{m} \lambda_{j}P_{j} \Biggr) \leq\sum _{j=1}^{m}\lambda_{j}f(P_{j}) $$

for all convex combinations of points $P_{j}\in\mathcal{S}$. An affine function $f:\operatorname{aff}\mathcal{S}\to\mathbb{R}$ satisfies the equality in equation (2) for all affine combinations of points $P_{j}\in\mathcal{S}$.

Throughout the paper, we use the *n*-dimensional space $\mathbb {X}=\mathbb{R}^{n}$ over the field $\mathbb{R}$.

## Convex functions on the simplex

The section is a review of the known results on the Hermite-Hadamard inequality for simplices, and it refers to its generic background. The main notification is concentrated in Lemma 2.1, which is also the generalization of the Hermite-Hadamard inequality.

Let $A_{1},\ldots,A_{n+1}\in\mathbb{R}^{n}$ be points so that the points $A_{1}-A_{n+1},\ldots,A_{n}-A_{n+1}$ are linearly independent. The convex hull of the points $A_{i}$ written in the form of $A_{1} \cdots A_{n+1}$ is called the *n*-simplex in $\mathbb{R}^{n}$, and the points $A_{i}$ are called the vertices. So, we use the denotation

3 $$ A_{1}\cdots A_{n+1}=\operatorname{conv} \{A_{1},\ldots,A_{n+1}\}. $$

The convex hull of *n* vertices is called the facet or $(n-1)$-face of the given *n*-simplex.

The analytic presentation of points of an *n*-simplex $\mathcal {A}=A_{1}\cdots A_{n+1}$ in $\mathbb{R}^{n}$ arises from the *n*-volume by means of the Lebesgue measure or the Riemann integral. We will use the abbreviation vol instead of $\operatorname{vol}_{n}$.

Let $A\in\mathcal{A}$ be a point, and let $\mathcal{A}_{i}$ be the convex hull of the set containing the point *A* and vertices $A_{j}$ for $j\neq i$, formally as

4 $$ \mathcal{A}_{i}=\operatorname{conv}\{A_{1}, \ldots,A_{i-1},A,A_{i+1},\ldots ,A_{n+1}\}. $$

Each $\mathcal{A}_{i}$ is a facet or *n*-subsimplex of $\mathcal{A}$, so $\operatorname{vol}(\mathcal{A}_{i})=0$ or $0<\operatorname{vol}(\mathcal {A}_{i})\leq\operatorname{vol}(\mathcal{A})$, respectively. The sets $\mathcal{A}_{i}$ satisfy $\mathcal{A}=\bigcup_{i=1}^{n+1}\mathcal{A}_{i}$ and $\operatorname{vol}(\mathcal{A}_{i}\cap\mathcal{A}_{j})=0$ for $i\neq j$, and so it follows that $\operatorname{vol}(\mathcal{A})=\sum_{i=1}^{n+1}\operatorname{vol}(\mathcal{A}_{i})$.

The point *A* can be uniquely represented as the convex combination of the vertices $A_{i}$ by means of

5 $$ A=\sum_{i=1}^{n+1} \alpha_{i}A_{i}, $$

where we have the coefficients

6 $$ \alpha_{i}=\frac{\operatorname{vol}(\mathcal{A}_{i})}{\operatorname{vol}(\mathcal{A})}. $$

If the point *A* belongs to the interior of the *n*-simplex $\mathcal {A}$, then all sets $\mathcal{A}_{i}$ are *n*-simplices, and consequently all coefficients $\alpha_{i}$ are positive. Furthermore, the reverse implications are valid.

If *μ* is a positive measure on $\mathbb{R}^{n}$, and if $\mathcal {S}\subseteq\mathbb{R}^{n}$ is a measurable set such that $\mu(\mathcal{S})>0$, then the integral mean point

7 $$ S= \biggl(\frac{\int_{\mathcal{S}}x_{1} \,d\mu(x)}{\mu(\mathcal {S})},\ldots, \frac{\int_{\mathcal{S}}x_{n} \,d\mu(x)}{\mu(\mathcal {S})} \biggr) $$

is called the *μ*-barycenter of the set $\mathcal{S}$. In the above integrals, points $x\in\mathcal{S}$ are used as $x=(x_{1},\ldots ,x_{n})$. The *μ*-barycenter *S* belongs to the convex hull of $\mathcal{S}$. When we use the Lebesgue measure, we say just barycenter. If $\mathcal{S}$ is closed and convex, then a *μ*-integrable continuous convex function $f:\mathcal{S}\to\mathbb{R}$ satisfies the inequality

8 $$ f \biggl(\frac{\int_{\mathcal{S}}x_{1} \,d\mu(x)}{\mu(\mathcal {S})},\ldots, \frac{\int_{\mathcal{S}}x_{n} \,d\mu(x)}{\mu(\mathcal {S})} \biggr) \leq \frac{\int_{\mathcal{S}}f(x) \,d\mu(x)}{\mu(\mathcal{S})} $$

as a special case of Jensen’s inequality for multivariate convex functions; see the excellent McShane paper in [1]. If *f* is affine, then the equality is valid in (8).

We consider a convex function *f* defined on the *n*-simplex $\mathcal {A}=A_{1}\cdots A_{n+1}$. The following lemma presents a basic inequality for a convex function on the simplex, and it refers to the connection of the simplex barycenter with simplex vertices.

### Lemma 2.1

*Let* *μ* *be a positive measure on* $\mathbb{R}^{n}$. *Let* $\mathcal {A}=A_{1}\cdots A_{n+1}$ *be an* *n*-*simplex in* $\mathbb{R}^{n}$ *such that* $\mu(\mathcal{A})>0$. *Let* *A* *be the* *μ*-*barycenter of* $\mathcal {A}$, *and let* $\sum_{i=1}^{n+1}\alpha_{i}A_{i}$ *be its unique convex combination by means of*

9 $$ A= \biggl(\frac{\int_{\mathcal{A}}x_{1} \,d\mu(x)}{\mu(\mathcal {A})},\ldots, \frac{\int_{\mathcal{A}}x_{n} \,d\mu(x)}{\mu(\mathcal {A})} \biggr) = \sum_{i=1}^{n+1}\alpha_{i}A_{i}. $$

*Then each convex function* $f:\mathcal{A}\to\mathbb{R}$ *satisfies the double inequality*

10 $$ f \Biggl(\sum_{i=1}^{n+1} \alpha_{i}A_{i} \Biggr)\leq \frac{\int_{\mathcal{A}}f(x) \,d\mu(x)}{\mu(\mathcal{A})} \leq\sum _{i=1}^{n+1}\alpha_{i}f(A_{i}). $$

### Proof

We have three cases depending on the position of the *μ*-barycenter *A* within the simplex $\mathcal{A}$.

If *A* is an interior point of $\mathcal{A}$, then we take a supporting hyperplane $x_{n+1}=h_{1}(x)$ at the graph point $(A,f(A))$, and the secant hyperplane $x_{n+1}=h_{2}(x)$ passing through the graph points $(A_{1},f(A_{1})),\ldots,(A_{n+1},f(A_{n+1}))$. Using the affinity of the functions $h_{1}$ and $h_{2}$, we get

11 $$\begin{aligned} f \Biggl(\sum_{i=1}^{n+1} \alpha_{i}A_{i} \Biggr) =& h_{1}(A) = \frac{\int_{\mathcal{A}}h_{1}(x) \,d\mu(x)}{\mu(\mathcal{A})} \\ \leq& \frac{\int_{\mathcal{A}}f(x) \,d\mu(x)}{\mu(\mathcal{A})} \\ \leq& \frac{\int_{\mathcal{A}}h_{2}(x) \,d\mu(x)}{\mu(\mathcal{A})} = h_{2}(A) \\ =& \sum_{i=1}^{n+1}\alpha_{i}h_{2}(A_{i}) = \sum_{i=1}^{n+1}\alpha_{i}f(A_{i}) \end{aligned}$$

because $h_{2}(A_{i})=f(A_{i})$. So, formula (10) works for the interior point *A*.

If *A* is a relative interior point of a certain *k*-face where $1 \leq k \leq n-1$, then we can apply the previous procedure to the respective *k*-simplex. For example, if $A_{1}\cdots A_{k+1}$ is the observed *k*-face, then the coefficients $\alpha_{1},\ldots,\alpha_{k+1}$ are positive, and the coefficients $\alpha_{k+2},\ldots,\alpha_{n+1}$ are equal to zero.

If *A* is a simplex vertex, suppose that $A=A_{1}$, then the trivial inequality $f(A_{1})\leq f(A_{1})\leq f(A_{1})$ represents formula (10). □

More generally, if the *μ*-barycenter *A* lies in the interior of $\mathcal{A}$, the inequality in formula (10) holds for all *μ*-integrable functions $f:\mathcal{A}\to\mathbb{R}$ that admit a supporting hyperplane at *A*, and satisfy the supporting-secant hyperplane inequality

12 $$ h_{1}(x) \leq f(x) \leq h_{2}(x) $$

for every point *x* of the simplex $\mathcal{A}$.

Lemma 2.1 was obtained in [2], Corollary 1, the case $\alpha_{i}=1/(n+1)$ was obtained in [3], Theorem 2, and a similar result was obtained in [4], Theorem 2.4.

By applying the Lebesgue measure or the Riemann integral in Lemma 2.1, the condition in (9) gives the barycenter

13 $$ A= \biggl(\frac{\int_{\mathcal{A}}x_{1} \,dx}{\operatorname{vol}(\mathcal {A})},\ldots, \frac{\int_{\mathcal{A}}x_{n} \,dx}{\operatorname{vol}(\mathcal{A})} \biggr) = \frac{\sum_{i=1}^{n+1}A_{i}}{n+1}, $$

and its use in formula (10) implies the Hermite-Hadamard inequality

14 $$ f \biggl(\frac{\sum_{i=1}^{n+1}A_{i}}{n+1} \biggr)\leq \frac{\int_{\mathcal{A}}f(x) \,dx}{\operatorname{vol}(\mathcal{A})} \leq \frac{\sum_{i=1}^{n+1}f(A_{i})}{n+1}. $$

The above inequality was introduced by Neuman in [5]. An approach to this inequality can be found in [6].

The discrete version of Lemma 2.1 contributes to the Jensen inequality on the simplex.

### Corollary 2.2

*Let* $\mathcal{A}=A_{1}\cdots A_{n+1}$ *be an* *n*-*simplex in* $\mathbb {R}^{n}$, *and let* $P_{1},\ldots,P_{m}\in\mathcal{A}$ *be points*. *Let* $A=\sum_{j=1}^{m}\lambda_{j}P_{j}$ *be a convex combination of the points* $P_{j}$, *and let* $\sum_{i=1}^{n+1}\alpha_{i}A_{i}$ *be the unique convex combination of the vertices* $A_{i}$ *such that*

15 $$ A=\sum_{j=1}^{m} \lambda_{j}P_{j}=\sum_{i=1}^{n+1} \alpha_{i}A_{i}. $$

*Then each convex function* $f:\mathcal{A}\to\mathbb{R}$ *satisfies the double inequality*

16 $$ f \Biggl(\sum_{i=1}^{n+1} \alpha_{i}A_{i} \Biggr) \leq\sum _{j=1}^{m}\lambda_{j}f(P_{j}) \leq\sum_{i=1}^{n+1}\alpha_{i}f(A_{i}). $$

### Proof

The discrete measure *μ* concentrated at the points $P_{j}$ by the rule

17 $$ \mu \bigl(\{P_{j}\} \bigr)=\lambda_{j} $$

can be utilized in Lemma 2.1 to obtain the discrete inequality in formula (16). □

Putting $\sum_{j=1}^{m}\lambda_{j}P_{j}$ instead of $\sum_{i=1}^{n+1}\alpha_{i}A_{i}$ within the first term of formula (16), we obtain the Jensen inequality extended to the right.

Corollary 2.2 in the case $\alpha_{i}=1/(n+1)$ was obtained in [3], Corollary 4.

One of the most influential results of the theory of convex functions is the Jensen inequality (see [7] and [8]), and among the most beautiful results is certainly the Hermite-Hadamard inequality (see [9] and [10]). A significant generalization of the Jensen inequality for multivariate convex functions can be found in [1]. Improvements of the Hermite-Hadamard inequality for univariate convex functions were obtained in [11]. As for the Hermite-Hadamard inequality for multivariate convex functions, one may refer to [2, 4, 5, 12–16], and [17].

## Main results

Throughout the section, we will use an *n*-simplex $\mathcal {A}=A_{1}\cdots A_{n+1}$ in the space $\mathbb{R}^{n}$, and its two *n*-subsimplices which will be denoted with $\mathcal{B}$ and $\mathcal{C}$.

Let $B_{i}$ stand for the barycenter of the facet of $\mathcal{A}$ not containing the vertex $A_{i}$ by

18 $$ B_{i}=\frac{\sum_{i \neq j=1}^{n+1}A_{j}}{n}, $$

and let $\mathcal{B}=B_{1}\cdots B_{n+1}$ be the *n*-simplex of the vertices $B_{i}$.

**Figure 1:**
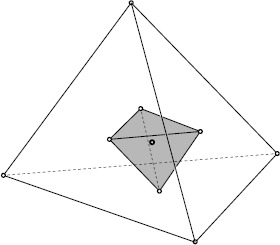
**The inscribed simplex as the barycenter extension.**

The simplices $\mathcal{A}$ and $\mathcal{B}$ in our three-dimensional space are tetrahedrons presented in Figure 1. Our aim is to extend the Hermite-Hadamard inequality to all points of the inscribed simplex $\mathcal{B}$ excepting its vertices. So, we focus on the non-peaked simplex $\mathcal{B}'=\mathcal{B}\setminus\{B_{1},\ldots,B_{n+1}\}$.

### Lemma 3.1

*Let* $\mathcal{A}=A_{1}\cdots A_{n+1}$ *be an* *n*-*simplex in* $\mathbb {R}^{n}$, *and let* $A=\sum_{i=1}^{n+1}\alpha_{i}A_{i}$ *be a convex combination of the vertices* $A_{i}$.

*The point* *A* *belongs to the* *n*-*simplex* $\mathcal{B}=B_{1}\cdots B_{n+1}$ *if and only if the coefficients* $\alpha_{i}$ *satisfy* $\alpha _{i}\leq1/n$.

*The point* *A* *belongs to the non*-*peaked simplex* $\mathcal{B}'=\mathcal {B}\setminus\{B_{1},\ldots,B_{n+1}\}$ *if and only if the coefficients* $\alpha_{i}$ *satisfy* $0<\alpha_{i}\leq1/n$.

### Proof

The first statement, relating to the simplex $\mathcal{B}$, will be covered as usual by proving two directions.

Let us assume that the coefficients $\alpha_{i}$ satisfy the limitations $\alpha_{i}\leq1/n$. Then the coefficients

19 $$ \beta_{i}=1-n\alpha_{i} $$

are nonnegative, and their sum is equal to 1. Since $\beta_{i}=1-\sum_{i \neq j=1}^{n+1}\beta_{j}$, the reverse connection

20 $$ \alpha_{i}=\frac{\sum_{i \neq j=1}^{n+1}\beta_{j}}{n} $$

follows. The last of the convex combinations

21 $$\begin{aligned} \begin{aligned}[b] A &= \sum_{i=1}^{n+1} \alpha_{i}A_{i} \\ &= \sum_{i=1}^{n+1}\frac{\sum_{i \neq j=1}^{n+1}\beta_{j}}{n}A_{i} =\sum_{i=1}^{n+1}\beta_{i} \frac{\sum_{i \neq j=1}^{n+1}A_{j}}{n} \\ &= \sum_{i=1}^{n+1}\beta_{i}B_{i} \end{aligned} \end{aligned}$$

confirms that the point *A* belongs to the simplex $\mathcal{B}$.

Let us assume that the point *A* belongs to the simplex $\mathcal{B}$. Then we have the convex combination $A=\sum_{i=1}^{n+1}\lambda_{i}B_{i}$. Using equation (21) in the reverse direction, we get the convex combinations equality

22 $$ \sum_{i=1}^{n+1} \lambda_{i}B_{i} =\sum_{i=1}^{n+1} \alpha_{i}A_{i} $$

with the coefficient connections $\alpha_{i}=\sum_{i\neq j=1}^{n+1}\lambda_{j}/n$ from which we may conclude that $\alpha_{i}\leq1/n$.

The second statement, relating to the non-peaked simplex $\mathcal {B}'$, follows from the first statement and the convex combinations in formula (18) which uniquely represent the facet barycenters $B_{i}$. □

We need another subsimplex of $\mathcal{A}$. Let *A* be a point belonging to the interior of $\mathcal{A}$. In this case, the sets $\mathcal{A}_{i}$ defined by formula (4) are *n*-simplices. Let $C_{i}$ stand for the barycenter of the simplex $\mathcal{A}_{i}$ by means of

23 $$ C_{i}=\frac{A+\sum_{i \neq j=1}^{n+1}A_{j}}{n+1}, $$

and let $\mathcal{C}=C_{1}\cdots C_{n+1}$ be the *n*-simplex of the vertices $C_{i}$.

### Lemma 3.2

*Let* $\mathcal{A}=A_{1}\cdots A_{n+1}$ *be an* *n*-*simplex in* $\mathbb {R}^{n}$, *and let* $A=\sum_{i=1}^{n+1}\alpha_{i}A_{i}$ *be a convex combination of the vertices* $A_{i}$ *with coefficients* $\alpha _{i}$ *satisfying* $\alpha_{i}>0$.

*The point* *A* *belongs to the non*-*peaked simplex* $\mathcal{C}'=\mathcal {C}\setminus\{C_{1},\ldots,C_{n+1}\}$ *if and only if the coefficients* $\alpha_{i}$ *satisfy the additional limitations* $\alpha_{i}\leq1/n$.

### Proof

Suppose that the coefficients $\alpha_{i}$ satisfy $0<\alpha_{i}\leq 1/n$. Let $\beta_{i}$ be the coefficients as in equation (19). Using the trivial equality $A=A/(n+1)+nA/(n+1)$, and the coefficient connections of equation (20), we get

24 $$\begin{aligned} A =& \sum_{i=1}^{n+1} \alpha_{i}A_{i} =\frac{1}{n+1}A +\frac{n}{n+1}\sum _{i=1}^{n+1}\alpha_{i}A_{i} \\ =& \sum_{i=1}^{n+1}\beta_{i} \frac{A}{n+1} +\sum_{i=1}^{n+1}\sum _{i \neq j=1}^{n+1}\beta_{j} \frac{A_{i}}{n+1} \\ =& \sum_{i=1}^{n+1}\beta_{i} \frac{A}{n+1} +\sum_{i=1}^{n+1} \beta_{i}\sum_{i \neq j=1}^{n+1} \frac{A_{j}}{n+1} \\ =& \sum_{i=1}^{n+1}\beta_{i} \frac{A+\sum_{i \neq j=1}^{n+1}A_{j}}{n+1} =\sum_{i=1}^{n+1} \beta_{i}C_{i} \end{aligned}$$

indicating that the point *A* lies in the simplex $\mathcal{C}$. To show that the convex combination $\sum_{i=1}^{n+1}\beta_{i}C_{i}$ does not represent any vertex, we will assume that some $\beta_{i_{0}}=1$. Then $\alpha_{i_{0}}=0$ as opposed to the assumption that all $\alpha _{i}$ are positive.

The proof of the reverse implication goes exactly in the same way as in the proof of Lemma 3.1. □

Each simplex $\mathcal{C}$ is homothetic to the simplex $\mathcal{B}$. Namely, combining equations (23) and (18), we can represent each vertex $C_{i}$ by the convex combination

25 $$ C_{i}=\frac{1}{n+1}A+\frac{n}{n+1}B_{i}. $$

Then it follows that

$$ C_{i}-A=\frac{n}{n+1}(B_{i}-A), $$

and using free vectors, we have the equality $\overrightarrow {AC_{i}}=(n/(n+1))\overrightarrow{AB_{i}}$. So, the simplices $\mathcal{C}$ and $\mathcal{B}$ are similar respecting the homothety with the center at *A* and the coefficient $n/(n+1)$.

If $A\in\mathcal{B}'$, then $\mathcal{C}\subset\mathcal{B}'$ by the convex combinations in formula (25). Combining Lemma 3.1 and Lemma 3.2, and applying Corollary 2.2 to the simplex inclusions $\mathcal{C}\subset \mathcal{B}$ and $\mathcal{B}\subset\mathcal{A}$, we get the Jensen type inequality as follows.

### Corollary 3.3

*Let* $\mathcal{A}=A_{1}\cdots A_{n+1}$ *be an* *n*-*simplex in* $\mathbb {R}^{n}$, *let* $A=\sum_{i=1}^{n+1}\alpha_{i}A_{i}$ *be a convex combination of the vertices* $A_{i}$ *with coefficients* $\alpha _{i}$ *satisfying* $0<\alpha_{i}\leq1/n$, *and let* $\beta_{i}=1-n\alpha_{i}$.

*Then it follows that*

26 $$ \sum_{i=1}^{n+1} \beta_{i}C_{i} = \sum_{i=1}^{n+1} \beta_{i}B_{i} = \sum_{i=1}^{n+1} \alpha_{i}A_{i}, $$

*and each convex function* $f:\mathcal{A}\to\mathbb{R}$ *satisfies the double inequality*

27 $$ \sum_{i=1}^{n+1} \beta_{i}f(C_{i}) \leq\sum_{i=1}^{n+1} \beta_{i}f(B_{i}) \leq\sum_{i=1}^{n+1} \alpha_{i}f(A_{i}). $$

The point *A* used in the previous corollary lies in the interior of the simplex $\mathcal{A}$ because the coefficients $\alpha_{i}$ are positive. In that case, the sets $\mathcal{A}_{i}$ are *n*-simplices, and they will be used in the main theorem that follows.

### Theorem 3.4

*Let* $\mathcal{A}=A_{1}\cdots A_{n+1}$ *be an* *n*-*simplex in* $\mathbb {R}^{n}$, *let* $A=\sum_{i=1}^{n+1}\alpha_{i}A_{i}$ *be a convex combination of the vertices* $A_{i}$ *with coefficients* $\alpha _{i}$ *satisfying* $0<\alpha_{i}\leq1/n$, *and let* $\beta_{i}=1-n\alpha_{i}$. *Let* $\mathcal{A}_{i}$ *be the simplices defined by formula* (4).

*Then each convex function* $f:\mathcal{A}\to\mathbb{R}$ *satisfies the double inequality*

28 $$ f \Biggl(\sum_{i=1}^{n+1} \alpha_{i}A_{i} \Biggr) \leq \sum _{i=1}^{n+1}\beta_{i} \frac{\int_{\mathcal{A}_{i}}f(x) \,dx}{ \operatorname{vol}(\mathcal{A}_{i})} \leq \sum_{i=1}^{n+1}\alpha_{i}f(A_{i}). $$

### Proof

Using the convex combinations equality $\sum_{i=1}^{n+1}\alpha _{i}A_{i}=\sum_{i=1}^{n+1}\beta_{i}C_{i}$, and applying the Jensen inequality to $f (\sum_{i=1}^{n+1}\beta _{i}C_{i} )$, we get

$$ f \Biggl(\sum_{i=1}^{n+1} \alpha_{i}A_{i} \Biggr) \leq \sum _{i=1}^{n+1}\beta_{i}f(C_{i}) = \sum_{i=1}^{n+1}\beta_{i}f \biggl( \frac{A+\sum_{i \neq j=1}^{n+1}A_{j}}{n+1} \biggr). $$

Summing the products of the Hermite-Hadamard inequalities for the function *f* on the simplices $\mathcal{A}_{i}$ and the coefficients $\beta_{i}$, it follows that

$$ \sum_{i=1}^{n+1} \beta_{i}f \biggl(\frac{A+\sum_{i \neq j=1}^{n+1}A_{j}}{n+1} \biggr) \leq \sum _{i=1}^{n+1}\beta_{i} \frac{\int_{\mathcal{A}_{i}}f(x) \,dx}{ \operatorname{vol}(\mathcal{A}_{i})} \leq \sum_{i=1}^{n+1}\beta_{i} \frac{f(A)+\sum_{i \neq j=1}^{n+1}f(A_{j})}{n+1}. $$

Repeating the procedure which was used for the derivation of formula (24), we obtain the series of equalities

$$\begin{aligned} \sum_{i=1}^{n+1} \beta_{i} \frac{f(A)+\sum_{i \neq j=1}^{n+1}f(A_{j})}{n+1} =& \frac{1}{n+1}f(A) + \frac{n}{n+1}\sum_{i=1}^{n+1} \beta_{i} \frac{\sum_{i \neq j=1}^{n+1}f(A_{j})}{n} \\ =& \frac{1}{n+1}f(A) +\frac{n}{n+1}\sum_{i=1}^{n+1} \frac{\sum_{i \neq j=1}^{n+ 1}\beta_{j}}{n}f(A_{i}) \\ =& \frac{1}{n+1}f \Biggl(\sum_{i=1}^{n+1} \alpha_{i}A_{i} \Biggr)+\frac {n}{n+1}\sum _{i=1}^{n+1}\alpha_{i}f(A_{i}). \end{aligned}$$

Finally, applying the Jensen inequality to $f (\sum_{i=1}^{n+1}\alpha_{i}A_{i} )$, we get the last inequality

$$ \frac{1}{n+1}f \Biggl(\sum_{i=1}^{n+1} \alpha_{i}A_{i} \Biggr)+\frac {n}{n+1}\sum _{i=1}^{n+1}\alpha_{i}f(A_{i}) \leq\sum_{i=1}^{n+1}\alpha_{i}f(A_{i}). $$

Bringing together all of the above, we obtain the multiple inequality

29 $$\begin{aligned} f \Biggl(\sum_{i=1}^{n+1} \alpha_{i}A_{i} \Biggr) \leq& \sum _{i=1}^{n+1}\beta_{i}f \biggl( \frac{A+\sum_{i \neq j=1}^{n+1}A_{j}}{n+1} \biggr) \leq \sum_{i=1}^{n+1} \beta_{i} \frac{\int_{\mathcal {A}_{i}}f(x) \,dx}{\operatorname{vol}(\mathcal{A}_{i})} \\ \leq& \sum_{i=1}^{n+1}\beta_{i} \frac{f(A)+\sum_{i \neq j=1}^{n+1}f(A_{j})}{n+1} \leq \sum_{i=1}^{n+1} \alpha_{i}f(A_{i}), \end{aligned}$$

of which the most important part is the double inequality in formula (28). □

The inequality in formula (29) is a generalization and refinement of the Hermite-Hadamard inequality. Taking the coefficients $\alpha_{i}=1/(n+1)$, in which case $\beta_{i}=1/(n+1)$, we realize the five terms inequality

30 $$\begin{aligned} f \biggl(\frac{\sum_{i=1}^{n+1}A_{i}}{n+1} \biggr) \leq& \frac{1}{n+1}\sum _{i=1}^{n+1}f \biggl(\frac{A_{i}+(n+2)\sum_{i \neq j=1}^{n+1}A_{j}}{(n+1)(n+1)} \biggr) \leq \frac{\int_{\mathcal{A}}f(x) \,dx}{\operatorname{vol}(\mathcal{A})} \\ \leq& \frac{1}{n+1}f \biggl(\frac{\sum_{i=1}^{n+1}A_{i}}{n+1} \biggr) +\frac{n}{n+1} \frac{\sum_{i=1}^{n+1}f(A_{i})}{n+1} \leq \frac{\sum_{i=1}^{n+1}f(A_{i})}{n+1}, \end{aligned}$$

where the second and fourth terms refine the Hermite-Hadamard inequality. The third term is generated from all of $n+1$ simplices $\mathcal {A}_{i}$. In the present case, these simplices have the same volume equal to $\operatorname{vol}(\mathcal{A})/(n+1)$.

The inequality in formula (30) excepting the second term was obtained in [2], Theorem 2. Similar inequalities concerning the standard *n*-simplex were obtained in [5, 6] and [18]. Special refinements of the left and right-hand side of the Hermite-Hadamard inequality were recently obtained in [19] and [20].

## Generalization to the function barycenter

If *μ* is a positive measure on $\mathbb{R}^{n}$, if $\mathcal {S}\subseteq\mathbb{R}^{n}$ is a measurable set, and if $g:\mathcal{S}\to\mathbb{R}$ is a nonnegative integrable function such that $\int_{\mathcal{S}}g(x) \,d\mu(x)>0$, then the integral mean point

31 $$ S= \biggl(\frac{\int_{\mathcal{S}}x_{1}g(x) \,d\mu(x)}{\int_{\mathcal {S}}g(x) \,d\mu(x)},\ldots, \frac{\int_{\mathcal{S}}x_{n}g(x) \,d\mu (x)}{\int_{\mathcal{S}}g(x) \,d\mu(x)} \biggr) $$

can be called the *μ*-barycenter of the function *g*. It is about the following measure. Introducing the measure *ν* as

32 $$ \nu(S)= \int_{\mathcal{S}}g(x) \,d\mu(x), $$

we get

33 $$ S= \biggl(\frac{\int_{\mathcal{S}}x_{1} \,d\nu(x)}{\nu(\mathcal {S})},\ldots, \frac{\int_{\mathcal{S}}x_{n} \,d\nu(x)}{\nu(\mathcal {S})} \biggr). $$

Thus the *μ*-barycenter of the function *g* coincides with the *ν*-barycenter of its domain $\mathcal{S}$. So, the barycenter *S* belongs to the convex hull of the set $\mathcal{S}$. By using the unit function $g(x)=1$ in formula (31), it is reduced to formula (7).

Utilizing the function barycenter instead of the set barycenter, we have the following reformulation of Lemma 2.1.

### Lemma 4.1

*Let* *μ* *be a positive measure on* $\mathbb{R}^{n}$. *Let* $\mathcal {A}=A_{1}\cdots A_{n+1}$ *be an* *n*-*simplex in* $\mathbb{R}^{n}$, *and let* $g:\mathcal{A}\to\mathbb{R}$ *be a nonnegative integrable function such that* $\int_{\mathcal{A}}g(x) \,d\mu(x)>0$. *Let* *A* *be the* *μ*-*barycenter of* *g*, *and let* $\sum_{i=1}^{n+1}\alpha_{i}A_{i}$ *be its unique convex combination by means of*

34 $$ A= \biggl(\frac{\int_{\mathcal{A}}x_{1}g(x) \,d\mu(x)}{\int_{\mathcal {A}}g(x) \,d\mu(x)},\ldots, \frac{\int_{\mathcal{A}}x_{n}g(x) \,d\mu (x)}{\int_{\mathcal{A}}g(x) \,d\mu(x)} \biggr) = \sum_{i=1}^{n+1}\alpha_{i}A_{i}. $$

*Then each convex function* $f:\mathcal{A}\to\mathbb{R}$ *satisfies the double inequality*

35 $$ f \Biggl(\sum_{i=1}^{n+1} \alpha_{i}A_{i} \Biggr) \leq \frac{\int_{\mathcal{A}}f(x)g(x) \,d\mu(x)}{ \int_{\mathcal{A}}g(x) \,d\mu(x)} \leq \sum _{i=1}^{n+1}\alpha_{i}f(A_{i}). $$

The proof of Lemma 2.1 can be employed as the proof of Lemma 4.1 by using the measure *ν* in formula (32) or by utilizing the affinity of the hyperplanes $h_{1}$ and $h_{2}$ in the form of the equalities

36 $$ h_{1,2} \biggl(\frac{\int_{\mathcal{A}}x_{1}g(x) \,d\mu(x)}{\int _{\mathcal{A}}g(x) \,d\mu(x)},\ldots, \frac{\int_{\mathcal {A}}x_{n}g(x) \,d\mu(x)}{\int_{\mathcal{A}}g(x) \,d\mu(x)} \biggr) =\frac{\int_{\mathcal{A}}h_{1,2}(x)g(x) \,d\mu(x)}{ \int_{\mathcal{A}}g(x) \,d\mu(x)}. $$

Lemma 4.1 is an extension of the Fejér inequality (see [21]) to multivariable convex functions. As regards univariable convex functions, using the Lebesgue measure on $\mathbb{R}$ and a closed interval as 1-simplex in Lemma 4.1, we get the following generalization of the Fejér inequality.

### Corollary 4.2

*Let* $[a,b]$ *be a closed interval in* $\mathbb{R}$, *and let* $g:[a,b]\to \mathbb{R}$ *be a nonnegative integrable function such that* $\int _{a}^{b}g(x) \,dx>0$. *Let* *c* *be the barycenter of* *g*, *and let* $\alpha a+\beta b$ *be its unique convex combination by means of*

37 $$ c=\frac{\int_{a}^{b}xg(x) \,dx}{\int_{a}^{b}g(x) \,dx}=\alpha a+\beta b. $$

*Then each convex function* $f:[a,b]\to\mathbb{R}$ *satisfies the double inequality*

38 $$ f(\alpha a+\beta b) \leq\frac{\int_{a}^{b}f(x)g(x) \,dx}{\int _{a}^{b}g(x) \,dx} \leq\alpha f(a)+\beta f(b). $$

Fejér used a nonnegative integrable function *g* that is symmetric with respect to the midpoint $c=(a+b)/2$. Such a function satisfies $g(x)=g(2c-x)$, and therefore

$$ \int_{a}^{b}(x-c)g(x) \,dx=0. $$

As a consequence it follows that

$$ \frac{\int_{a}^{b}xg(x) \,dx}{\int_{a}^{b}g(x) \,dx} =\frac{\int_{a}^{b}(x-c)g(x) \,dx}{\int_{a}^{b}g(x) \,dx} +\frac{\int_{a}^{b}cg(x) \,dx}{\int_{a}^{b}g(x) \,dx}= \frac{a+b}{2}, $$

and formula (38) with $\alpha=\beta=1/2$ turns into the Fejér inequality

39 $$ f \biggl(\frac{a+b}{2} \biggr) \leq\frac{\int_{a}^{b}f(x)g(x) \,dx}{\int_{a}^{b}g(x) \,dx} \leq \frac{f(a)+f(b)}{2}. $$

Using the barycenters of the restrictions of *g* onto simplices $\mathcal{A}_{i}$ in formula (4), we have the following generalization of Theorem 3.4.

### Theorem 4.3

*Let* *μ* *be a positive measure on* $\mathbb{R}^{n}$. *Let* $\mathcal {A}=A_{1}\cdots A_{n+1}$ *be an* *n*-*simplex in* $\mathbb{R}^{n}$, *let* $A=\sum_{i=1}^{n+1}\alpha_{i}A_{i}$ *be a convex combination of the vertices* $A_{i}$ *with coefficients* $\alpha_{i}$ *satisfying* $0<\alpha _{i}\leq1/n$, *and let* $\beta_{i}=1-n\alpha_{i}$. *Let* $\mathcal{A}_{i}$ *be the simplices defined by formula* (4), *and let* $g_{i}:\mathcal{A}_{i}\to\mathbb{R}$ *be nonnegative integrable functions such that* $\int_{\mathcal{A}_{i}}g_{i}(x) \,d\mu (x)>0$ *and*

40 $$ C_{i}= \biggl(\frac{\int_{\mathcal{A}_{i}}x_{1}g_{i}(x) \,d\mu(x)}{\int _{\mathcal{A}_{i}}g_{i}(x) \,d\mu(x)},\ldots, \frac{\int_{\mathcal {A}_{i}}x_{n}g_{i}(x) \,d\mu(x)}{\int_{\mathcal{A}_{i}}g_{i}(x) \,d\mu (x)} \biggr) =\frac{A+\sum_{i \neq j=1}^{n+1}A_{j}}{n+1}. $$

*Then each convex function* $f:\mathcal{A}\to\mathbb{R}$ *satisfies the double inequality*

41 $$ f \Biggl(\sum_{i=1}^{n+1} \alpha_{i}A_{i} \Biggr) \leq \sum _{i=1}^{n+1}\beta_{i} \frac{\int_{\mathcal{A}_{i}}f(x)g_{i}(x) \,d\mu(x)}{ \int_{\mathcal{A}_{i}}g_{i}(x) \,d\mu(x)} \leq \sum_{i=1}^{n+1}\alpha_{i}f(A_{i}). $$

### Proof

The first step of the proof is to apply Lemma 4.1 to the functions *f* and $g_{i}$ on the simplex $\mathcal{A}_{i}$ in the way of

$$ f \biggl(\frac{A+\sum_{i \neq j=1}^{n+1}A_{j}}{n+1} \biggr) \leq \frac{\int_{\mathcal{A}_{i}}f(x)g_{i}(x) \,d\mu(x)}{\int_{\mathcal {A}_{i}}g_{i}(x) \,d\mu(x)} \leq \frac{f(A)+\sum_{i \neq j=1}^{n+1}f(A_{j})}{n+1}. $$

Summing the products of the above inequalities with the coefficients $\beta_{i}$, we obtain the double inequality that may be combined with formula (29), and so we obtain the multiple inequality

42 $$\begin{aligned} f \Biggl(\sum_{i=1}^{n+1} \alpha_{i}A_{i} \Biggr) \leq& \sum _{i=1}^{n+1}\beta_{i}f \biggl( \frac{A+\sum_{i \neq j=1}^{n+1}A_{j}}{n+1} \biggr) \leq \sum_{i=1}^{n+1} \beta_{i} \frac{\int_{\mathcal{A}_{i}}f(x)g_{i}(x) \,d\mu(x)}{\int_{\mathcal{A}_{i}}g_{i}(x) \,d\mu(x)} \\ \leq& \sum_{i=1}^{n+1}\beta_{i} \frac{f(A)+\sum_{i \neq j=1}^{n+1}f(A_{j})}{n+1} \leq \sum_{i=1}^{n+1} \alpha_{i}f(A_{i}) \end{aligned}$$

containing the double inequality in formula (41). □

The conditions in formula (40) require that the *μ*-barycenter of the function $g_{i}$ coincides with the barycenter $C_{i}= (A+\sum_{i \neq j=1}^{n+1}A_{j} )/(n+1)$ of the simplex $\mathcal{A}_{i}$.

Using the Lebesgue measure and functions $g_{i}(x)=1$, the inequality in formula (42) reduces to the inequality in formula (29).
